# Intraoperative Utilization of Indocyanine Green (ICG) Dye for the Assessment of Ovarian Perfusion—Case Report and Review of the Literature

**DOI:** 10.3390/jcm12185923

**Published:** 2023-09-12

**Authors:** Ruben Plöger, Mateja Condic, Damian J. Ralser, Hannah M. Plöger, Eva K. Egger, Lucia A. Otten, Alexander Mustea

**Affiliations:** 1 Department of Gynecology and Gynecological Oncology, University Hospital Bonn, 53127 Bonn, Germany; mateja.condic@ukbonn.de (M.C.); damian.ralser@ukbonn.de (D.J.R.); eva-katharina.egger@ukbonn.de (E.K.E.); lucia.otten@ukbonn.de (L.A.O.); alexander.mustea@ukbonn.de (A.M.); 2 Department of Paediatrics, University Hospital Bonn, 53127 Bonn, Germany; hannah.ploeger@ukbonn.de

**Keywords:** ovarian torsion, oophorectomy, indocyanine green, fluorescence guides surgery, adnexal torsion

## Abstract

The assessment of ovarian perfusion after detorsion is crucial in the surgical management of patients with ovarian torsion. In current routine clinical practice, the surgical decision (preservation of the ovary versus oophorectomy) is based on the subjective impression of the surgeon. Intraoperative indocyanine green (ICG) angiography has been shown to sufficiently reflect tissue perfusion with a potential impact on the surgical procedure. Currently, there are only sparse data available on the utilization of ICG in the surgical treatment of ovarian torsion. Here, we describe the successful intraoperative use of ICG in a 17-year-old female patient with ovarian torsion who underwent ovary-preserving surgery. Further, a systematic literature review was performed. Based on the data available to date, the use of ICG in the surgical treatment of ovarian torsion is feasible and safe. The extent to which this might reduce the necessity for oophorectomy has to be evaluated in further investigations.

## 1. Introduction

Ovarian torsion is a rarely occurring gynecological emergency leading to an impairment of ovarian perfusion with consecutive hemorrhagic infarction and ovarian necrosis. This condition requires rapid surgical treatment by means of detorquing the twisted ovary to restore perfusion and thus to preserve ovarian function. The incidence is difficult to estimate, but the fact that ovarian torsion is sometimes defined as the fifth most common surgical emergency or that ovarian torsion accounts for 2.7% of all surgical emergencies [1] confirms that it is a highly relevant illness. Patient groups associated with ovarian torsion include women undergoing fertilization treatments [2]. Several case reports of ovarian torsion in pregnant patients and in the pediatric population [3,4,5,6] have been published. Furthermore, several studies reveal that right-sided torsions are more common [7,8]. Although some case reports describe conservative treatments [9], the most common treatment is surgical. Based on the location and time period of the published study, the main treatment methods differ between a laparoscopic approach and an approach with laparotomy [10,11]. In the developed world, the laparoscopic approach is currently the most common. The intraoperative assessment of restored ovarian perfusion is crucial for the surgical approach (ovarian preservation versus oophorectomy); however, the intraoperative decision is based on the subjective impression of the surgeon [12,13]. In this regard, a black or blue color and/or an enlargement of the ovary signify interrupted blood flow, whereas a regression of the ovarian size and—in the case of acute torsion—regained ovarian color represent signs of successful restoration of ovarian blood flow. These visual features of re-established ovarian perfusion might develop—especially after prolonged torsion—with delay, thereby leading to misjudgment and consequential oophorectomy. Therefore, alternative assessment methods are necessary to support decision making. 

Laboratory findings proving the restoration of ovarian blood flow have not yet been discovered. Radiologic features indicating ovarian torsion have been described and could support diagnostic and therapeutic decision making, always in combination with clinical signs such as severe pain, nausea, vomiting, and adnexal tenderness. In B-mode during an ultrasound examination, ultrasound features of ovarian torsion [14] include evidence of an enlarged ovary, ovarian edema, minimal free fluid, shift in the ovarian position toward the midline, variable echogenicity as a sign of cystic or hemorrhagic degeneration in the case of a long-standing ovarian infarction, and a follicular ring sign [15]. These signs often coincide with the existence of risk factors, also determined using ultrasound in B-mode, which are large cystic ovaries, such as after hyperstimulation [16], or the presence of an ovarian mass between 5 and 10 cm [17]. However, these features take time to normalize after the restoration of ovarian blood flow. Doppler ultrasound enables the illustration of blood flow and thus provides an almost immediate proof after restored perfusion. The absence of arterial flow is a sign of poor prognosis [14], and the lack of ovarian venous flow shows a sensitivity of 100% and specificity of 97% for ovarian torsion [18]. In 13–88% of patients with ovarian torsion, the whirlpool sign appears to be caused by the swirling blood flow in the twisted ovarian pedicle [19,20,21]. This pathognomonic sign of ovarian torsion is also seen in one third of patients on CT or MRI [10,22]. Using CT and MRI, a subacute ovarian hematoma or an abnormal or absent ovarian enhancement are diagnosed more easily than by using ultrasound [23], while evidence of enlarged or shifted ovaries in CT and MRI, as signs of ovarian torsion, is seen with a similar precision as that achieved with ultrasound. Radiographic diagnostic tools such as ultrasound, MRI and CT provide the possibility of a conservative treatment of ovarian torsion, for example, when an ultrasound-guided aspiration of the ovarian cyst is performed, resulting in spontaneous detorsion of the ovary [9]. However, an intraoperative, real-time assessment tool to prove the successful restoration of ovarian blood flow is needed. 

Indocyanine green (ICG) angiography is used across various medical fields for evaluation of tissue perfusion [24]. In gynecology [25], ICG is applied for visualization of the vascular perfusion of the vaginal cuff after total hysterectomy [26,27], of the ureteral course [28], and of endometriosis [29,30], as well as for detection of the sentinel lymph node [31,32,33]. The visualization of vascular perfusion of the vaginal cuff using ICG is feasible and complication-free but with unclear clinical profit, while a more objective analysis of its fluorescence has been established in colorectal surgery by applying the correlation between fluorescence and leakage of the colorectal anastomosis to determine further surgical steps [34]. This kind of correlation between vascular perfusion marked by ICG and the vaginal cuff dehiscence may allow a reduction in dehiscence rates in the future, which currently range between 0.64% and 1.35% [35]. The use of ICG to prevent iatrogenic ureteral injury via real-time delineation of the ureter has the advantage that only the tip of the ureteral catheter has to be inserted and a further intervention for the insertion for a ureteral stent is avoided. [36]. In the case of surgical management of endometriosis, ICG allows for a detection of the polymorphic-appearing endometriosis lesions based on their neovascularization, but its usefulness is inconsistent, as shown in a systematic review [37]. Furthermore, ICG is used for sentinel lymph node mapping after the preoperative lymphoscintigraphy in the diagnostics of breast cancer [38]. Therefore, the use of ICG for sentinel lymph node identification is superior to the established combined use of a radioactive tracer and a blue dye in regard to the logistical challenges between the operating room and radiology, its excellent safety profile without radiopharmaceutical material, with good tissue penetration, and with real-time intraoperative imaging capabilities [39,40]. Recently, a higher sentinel lymph node detection rate in breast surgery though the use of ICG compared to radio-guided surgery using radioisotope technetium, sometimes combined with blue dye, was shown [33]. Thus, ICG is a safe and effective alternative to technetium in breast surgery [33,41]. The use of ICG in sentinel diagnosis is documented in further gynecologic areas such as in the treatment of vulva cancer [32,42], in cervical cancer [32] and in endometrial cancer [43]. While various safe application possibilities of ICG dye have been demonstrated, its application in the treatment of ovarian torsion has not yet been established; however, it is promising to analyze ovarian perfusion after detorsion. Here, we describe the successful intraoperative use of ICG in a 17-year-old female patient with ovarian torsion who underwent ovary-preserving surgery. Further, a systematic literature review was performed. 

## 2. Materials and Methods

**Figure 1:**
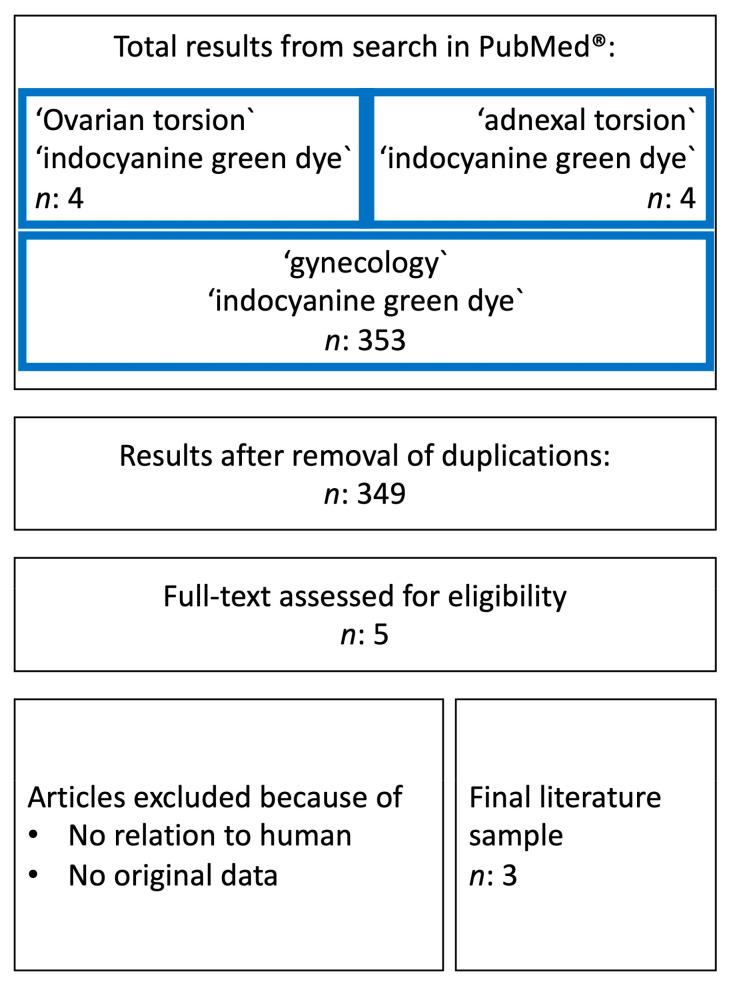
Flowchart of the PubMed search. *n*: number.

The patient presented herself emergently during the night shift. She consented to the treatment and to the publication of the case. Her parents were informed and agreed as well. The literature search was performed using the PubMed database. Studies that were published until October 2022 were considered. The following terms were applied: ‘Ovarian torsion’ and ‘ICG dye’, ‘adnexal torsion’ and ‘ICG dye’ and ‘gynecology’ and ‘ICG dye’. Duplications were removed. The title and abstract of the retrieved publications were read to assess their relevance. Publications with promising abstracts were full-text assessed for eligibility. Study design and language were not restricted (Figure 1).

## 3. Results

### 3.1. Case Report

**Figure 2:**
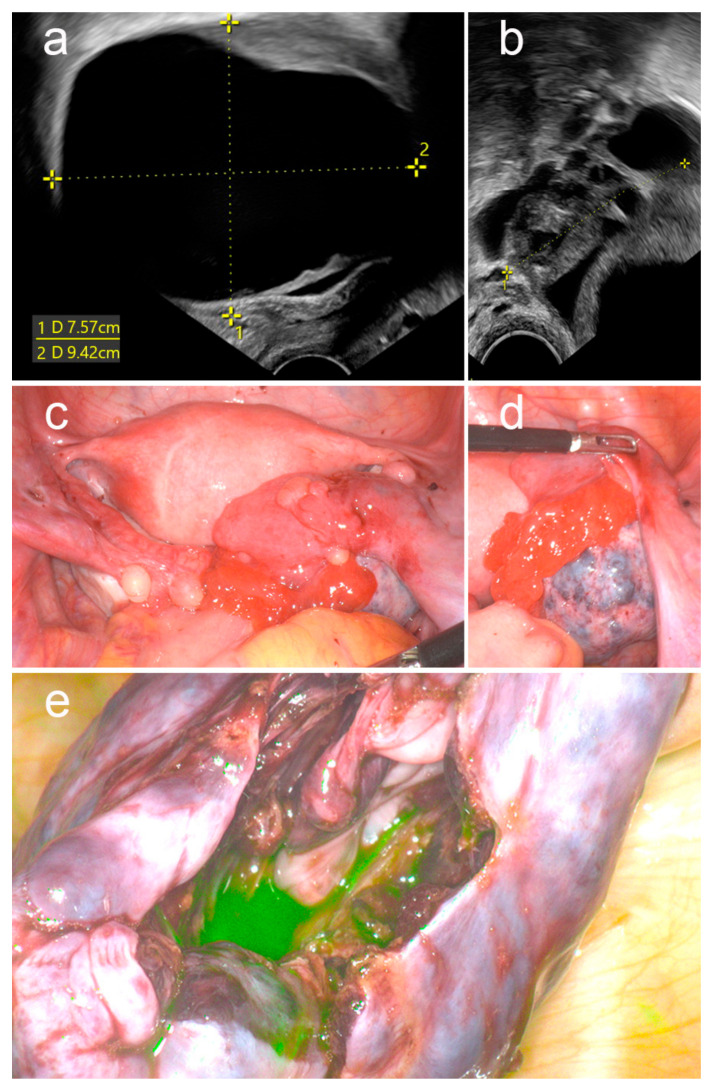
Ultrasonic (**a**,**b**) and intraoperative (**c**–**e**) presentation of the right ovary. The right ovary shows an ovarian cyst measuring 75.2 × 94.2 mm (**a**) with ovarian stromal edema and edema around the ovary (**b**). Intraoperatively, the right ovary appears lived and ischemic (**c**,**d**). Following detorsion and cystectomy, ICG angiography demonstrated restored ovarian perfusion (**e**). The photos were taken with the pinpoint endoscopic fluorescence imaging camera system (Stryker^®^, Duisburg, Germany).

A 17-year-old female patient presented with severe, sudden onset of pain in the right lower abdomen. Furthermore, she suffered from pronounced nausea with repeated vomiting. The patient’s history revealed the presence of a right ovarian cyst that was diagnosed several years ago. The rest of the patient’s history was unremarkable. Blood tests showed no abnormalities except for mild leukocytosis (12.56 G/L, normal range: 4–10 G/L). Serum beta HCG and C-reactive protein were in normal range. The clinical examination revealed severe abdominal tenderness with localized tenderness in the right lower abdomen. Transvaginal ultrasound showed a cyst on the right ovary measuring 75 × 94 mm with the presence of ovarian stromal edema (Figure 2a,b) highly suspicious for right-sided ovarian torsion. Hence, laparoscopic surgery was conducted, which confirmed torsion of the right ovary (Figure 2c,d). Intraoperatively, the right ovary was livid and ischemic. The rest of the situs was normal. Detorsion and enucleation of the ovarian cyst were performed. Following detorsion, the ovary remained livid with no evidence of recovery. To assess the ovarian perfusion more sufficiently, ICG dye (Diagnostic Green^®^, Aschheim-Dornach, Germany) was applied intravenously (2 mL equivalent to 10 mg). ICG angiography using an endoscopic fluorescence imaging system (Stryker^®^, Duisburg, Germany) demonstrated restored ovarian perfusion (Figure 2e). Histopathological examination revealed a dermoid cyst. No signs of necrosis were reported. The postoperative course was unremarkable. The drainage could be removed on the first postoperative day and the patient was discharged on the second postoperative day. At 6 months of follow-up, there was no evidence of secondary ovarian necrosis or infection.

### 3.2. Use of Indocyanine Green Dye

A systematic literature review (Figure 1 and Table 1) on ICG application for the evaluation of ovarian perfusion in the context of ovarian torsion was performed. There was no evidence for adverse events resulting from ICG application in *n* = 34 described cases [44,45,46]. Intraoperative ICG administration is reported to be feasible and its implementation in the treatment approach of ovarian torsion is unproblematic [44,46] given the already established use of ICG in other gynecological indications (s. above). Furthermore, Esposito et al. (2022) indicated that the use of the dye reduces surgery time. In five reported cases, a lack of ovarian perfusion on ICG angiography led to oophorectomy. In one case, necrosis was detected histopathologically [44]. The visualization of ICG perfusion was detected in a median time of 1 min [44]. The reported cases collectively demonstrate that the intraoperative utilization of ICG is beneficial in deciding whether to perform oophorectomy or to preserve the ovary [45,46]. In one study [46], ovary sparing based on the intraoperative use of ICG resulted in no long-term complications, as yearly follow-up ultrasound examinations showed normal ovaries with no evidence of pathologies. The high cost of the equipment needed in laparoscopy to use the ICG system is referred to as one of the main limitations [46]. 

## 4. Discussion

Ovarian torsion is commonly associated with younger age, as this case demonstrates [47]. This stresses that the indication for oophorectomy should be considered critically. The presented case displays the established risk factors for ovarian torsion such as an enlarged ovary (>5 cm) and presentation with typical clinical findings such as nausea, vomiting, and sudden onset of pain (s. above). Ultrasound findings were suggestive of ovarian torsion (ovarian stromal edema, presence of whirlpool sign) based on the known signs (see above, [21,48]). The right-sided bias of ovarian torsion [7,8] verified itself in this case. In the presented case, the application of ICG with consecutive visualization of restored ovarian perfusion prevented oophorectomy without signs of complications.

The application of ICG in the surgical treatment for ovarian torsion is documented in *n* = 14 cases of ovarian torsion in adult patients [44,45] and in *n* = 20 cases of ovarian torsion in children [46]. The low number of published case reports contrasts markedly with the prevalence of ovarian torsion [1] and thus demonstrates the low establishment rate of ICG in the treatment of ovarian torsion. In five cases, oophorectomy was performed because of the absence of ovarian perfusion in ICG angiography [44,46]. No complications were reported in the *n* = 33 cases with ovarian preservation. In only one case where oophorectomy was performed did the histopathological results report no evidence of ovarian necrosis [44]. However, due to the limited number of cases, a final evaluation of sensitivity and specificity cannot be carried out. Supportive data for the high sensitivity of ICG for ovarian necrosis are provided by a study based on a murine model. In this study, fluorescence intensity was shown to reliably predict ovarian necrosis [49]. Data on the future function of the ovary, such as the development of follicles or the level of anti-Müllerian hormone, are not reported and should be implemented in further investigations. 

The amount of dye used differs in the reported cases (Table 1); however, 5 to 10 mg of ICG was applied in the majority of cases, which is in line with the recommendations by one ICG manufacturer [50]. The effect of ICG—in the case of re-perfusion—is reported to appear in a median time of 1 min [44] and suits the visualization of anatomic structures, such as in the testis though ICG application between 30 and 60 s [51]. Esposito et al. argue that ICG proves to be very useful for the assessment of the ovary’s ischemic damage and of its re-perfusion, and thus for the decision making for or against an oophorectomy [46]. Further intraoperative decision aids may include the regression of ultrasound-detected absence of arterial flow, a lack of ovarian venous flow, and a whirlpool sign demonstrating the successful reperfusion of the ovary. However, intraoperative ultrasound lacks practicability based on the fact that the common laparoscopic approach requires the inflation of carbon dioxide gas, which limits ultrasound quality and visibility [52]. While CT and MRI are helpful diagnostic tools to evaluate perfusion in general, their intraoperative application to demonstrate the restoration of ovarian blood flow is irrelevant in most cases, as CT and MRI are rarely encountered in the surgery theater. Therefore, ICG may represent the only diagnostic tool to show reperfusion during surgery with the potential to reduce the rate of oophorectomy in patients with ovarian torsion. Postoperative histopathological evaluations of removed ovaries in patients with ovarian torsion confirm the presence of ischemia in only 43% [53,54] of cases, demonstrating the urgent need for a reliable diagnostic tool to support the sparing of the ovary. The resilience of ovaries argues for a general ovary-sparing technique in every case. There is increasing evidence for an ongoing hormonal ovarian function even in cases where ischemia is histologically confirmed [54]. On the contrary, the development of acute inflammation due to secondary ovarian necrosis may be a complication of ovarian sparing. Few case reports discuss the release of cytokines following ovarian necrosis as a reason for the death of infants after ovarian torsion [55,56]. Therefore, the sparing of every ovary after detorsion combined with the expectation of recovery may be harmful for the patient and lead to further surgical procedures. In order to differentiate these cases, the preoperative level of C-reactive protein may be useful, as it correlates with the necrosis of the ovary [57,58]. However, its use for the perfusion evaluation after detorsion is limited as it maintains a high level even after the successful restoration of ovarian blood flow and due to its long plasma half-life of about 19 h [59]. In conclusion, an intraoperative ICG-based evaluation of ovarian perfusion, in addition to a visual assessment carried out by the surgeon, represents a potential diagnostic tool to guide the intraoperative procedure (ovarian preservation versus no ovarian preservation). The extent to which secondary complications (re-operation, secondary inflammation, and limited fertility) could be diminished by the use of this technique, as opposed to general ovarian preservation, needs to be studied prospectively in larger collectives. The high cost can be shared either through the application of ICG in other fields of gynecological surgery [26,28,29,30] or in other disciplines [34,51]. These data and this case show an interesting new field of ICG’s application in gynecology. 

## 5. Conclusions

Based on the data available to date, the use of ICG in the surgical treatment of ovarian torsion is feasible and safe. The extent to which this might reduce the necessity for oophorectomy has to be evaluated in further investigations. 

## Figures and Tables

**Table 1:** Results of the literature review.

	Cases (*n*)	Absent Perfusion (*n*)	Oophorectomy (*n*)	Histologic Confirmed Necrosis (*n*)	Surgery Time (min)	Amount of ICG Dye
Nicholson et al., 2022 [44]	12	2	2	1	74	n. r.
Klar et al., 2022 [45]	1	0	0	0	n. r.	5 mg
Esposito et al., 2022 [46]	20	n. r.	3	n. r.	39.2	n. r.
Present case	1	0	0	0	110	10 mg (2 mL)

*n*: number, n. r.: not reported.

## Data Availability

Data available upon request.
